# FREE TRANSPORTATION SERVICE RECIPIENTS MAY HAVE BETTER QUALITY OF LIFE BUT STILL LACK IN IMPROVING HEALTH

**DOI:** 10.1093/geroni/igz038.624

**Published:** 2019-11-08

**Authors:** Machiko R Tomita

**Affiliations:** University at Buffalo, Buffalo, New York, United States

## Abstract

This study aimed to determine if service recipients (SRs) of free transportation services experience better quality of life, health, and function compared to pre-service recipients (PSRs). We conducted a cross-sectional study using personal interviews with 43 PSRs and 30 SRs belonged to a volunteer organization. Outcome measures were Older People’s Quality of Life (QoL), Center for Epidemiology Study-Depression, and Instrumental Activities of Daily Living (IADL). Total sample (N=73) had a mean age of 78.5 years and mostly female (86.3%). The majority of PSRs wanted to go to Drs’ offices (74.4%) and Grocery stores (60.5%), followed by Drug stores (44.2%), when the service becomes available. The figures were substantially smaller among SR (40.0%, 30%, and 13.3%, respectively). In PSRs, 67.4% expected to improve health once they start receiving the service, and 70. 0% of SRs said it did with the service. Using independent t-tests, SRs were significantly better in depression (p<.001), IADL (p=0.29) and most QoL items (life overall, social relationship, home and neighborhood, psychological and emotional well-being and leisure and activities; p=.047-p=.001), except for perceived health and finance. SRs (100%) were very satisfied with the service and drivers, but 80% of SRs said they wished to use more driving services than the allowable four times per month maximum. This limitation was due to the insufficient number of volunteers compared to a large number of people in need. Availability of more volunteer drivers will likely improve SRs health. Effective approaches to increase the number of driving volunteers are necessary.

